# Sickle Cell Anemia Associated With Increased In-Hospital Mortality in Post-Cardiac Arrest Patients

**DOI:** 10.7759/cureus.37987

**Published:** 2023-04-22

**Authors:** Mohamed Zakee Mohamed Jiffry, Rehana Hassan, Alexis Davis, Shelbie Scharf, Thilini Walgamage, Mohammad A Ahmed-Khan, Mehndi Dandwani

**Affiliations:** 1 Internal Medicine, Danbury Hospital, Danbury, USA; 2 School of Medicine, American University of the Caribbean, Cupecoy, SXM; 3 Department of Internal Medicine, University of Vermont, Burlington, USA

**Keywords:** sickle cell anaemia, in hospital mortality, in hospital cardiac arrest, sickle-cell disease, sickle cell trait

## Abstract

Introduction

Sickle cell anemia (SCA) is a hemoglobinopathy that arises from a point mutation in the beta-globin gene, which causes the polymerization of deoxygenated hemoglobin that leads to a wide variety of clinical complications. Deaths in patients with SCA most commonly arise from renal, cardiovascular disease, infections, and stroke. In-hospital cardiac arrest has been found to be more common in older patients and those on ventilatory life support, among others. This study aims to provide more insight into how SCA affects the risk of in-hospital mortality in post-cardiac arrest patients.

Methods

The National Inpatient Survey database years 2016 to 2019 was utilized. The International Classification of Diseases, Tenth Revision, Procedure Coding System (ICD-10 PCS) codes for cardiopulmonary resuscitation were used to identify in-hospital cardiac arrest (IHCA) patients. ICD-10 Clinical Modification (CM) codes were used to identify SCA and other medical comorbidities. Categorical data was compared using Person's chi-square test, and continuous variables were compared using the independent samples t-test. Multinomial logistic regression was used to study the effects of SCA on post-arrest in-hospital mortality controlling for age, Charlson comorbidity score, and demographic variables. Binomial logistic regression models for dichotomous variables were utilized in the subgroup and secondary outcomes analysis.

Results

In patients with IHCA, patients who had SCA were found to have a significantly increased risk of in-hospital mortality adjusted for baseline characteristics and Charlson comorbidity score (OR: 1.16, 95% CI: 1.02-1.32, p=0.0025). Patient characteristics most strongly associated with an increased risk of in-hospital mortality in this cohort were found to be Black race (OR: 1.92, 95% CI: 1.87-1.97, p<0.001) and self-payer status (OR: 2.14, 95% CI: 2.06-2.22, p<0.001). Subgroup analysis revealed only patients with sickle cell disease had a statistically significant increased risk of in-hospital mortality in this cohort (OR: 4.41, 95% CI: 3.5-5.55, p<0.001), and patients with sickle cell trait did not.

Conclusion

In patients with IHCA, SCA is associated with an increased risk of in-hospital mortality. This risk was confined to patients with sickle cell disease and not patients with sickle cell trait.

## Introduction

Sickle cell anemia (SCA) is a term that encompasses several genotypes, all of which follow Mendelian inheritance. This hereditary hemoglobinopathy arises from a point mutation in B-globin which causes a single amino acid substitution [1]. The resulting disease subgroups include sickle cell disease (HbSS), sickle cell - hemoglobin C disease (HbSC), and beta-zero thalassemia (HbB0thal)/beta-plus thalassemia (HbB+thal), among other sickle cell disorders. B-globin gene mutations cause polymerization of hemoglobin when deoxygenated, and this polymerization leads to sickling of the normal red blood cell shape, lending itself to a wide presentation of clinical complications [1]. Inpatient management of acute complications of sickle cell anemia includes vascular occlusion events, severe anemia, and now less commonly, invasive infections. Acute cardiac manifestations of SCA include myocardial infarction, dysrhythmia, sudden cardiac death, and dysfunction, and acute pulmonary manifestations of SCA include acute chest syndrome, asthma exacerbations, and pulmonary embolism. While chronic organ failure presents later in life and is a major cause of mortality among older SCA patients, sudden death due to acute complications remains a predominant cause of fatality throughout both earlier and later years [2]. 

Deaths in the older SCA population were shown in one study to arise most commonly from chronic complications of SCA, which include renal and cardiovascular disorders, whereas SCA-related deaths in the younger population have been associated with acute causes of infection and stroke. Additionally, SCA-related death rates have increased in the older population but have shown a decline in the younger population. When comparing factors that influence mortality trends in SCA-related deaths, it has been shown that specific interventions, such as penicillin prophylaxis and the pneumococcal conjugate vaccine, likely decreased the SCA-related deaths resulting from infection [3]. Although this intervention has proven favorable in the younger SCA population, there are limited interventions in the older SCA population once chronic organ damage has begun. Current research supports there being significant differences in life expectancies when the levels of specific types of hemoglobin are measured, with high levels of fetal hemoglobin being predictive of improved survival and increased adult life expectancy [4].

In-hospital cardiac arrest (IHCA) is defined as circulatory collapse followed by chest compressions and/or defibrillation and should be distinguished from sudden cardiac death without any resuscitation measures [5]. Looking at risk factors for IHCA in general, several have been proven to present a higher risk for poor patient outcomes, including old age, ventilator support, and the use of vasoactive agents [6]. The key factors playing into improved post-resuscitation care that improve patient outcomes following a return of spontaneous circulation (ROSC) are assessing/stabilizing the patient's cardiopulmonary status, determining the etiology of the arrest, neuroprotection, and preventing arrest recurrence [7].

This study aims to provide more insight into whether patients with SCA have increased mortality rates post-cardiac arrest during hospitalization than patients without SCA.

## Materials and methods

We planned to conduct an observational retrospective cohort study utilizing the National Inpatient Sample (NIS) database years 2016 to 2019. The databases combined data on over 28 million weighted hospital discharges. Data was extracted by utilizing the International Classification of Diseases, Tenth Revision (ICD-10) codes. As we utilized a publicly available deidentified administrative database, consent was not required, and IRB approval was waived.

For this study, the inclusion criteria were patients with and without sickle cell anemia who had an in-hospital cardiac arrest (IHCA) event. Patients who had an IHCA were identified by procedure-specific billing codes for cardiopulmonary resuscitation.

Exclusion criteria for this study were patients with concomitant anemia due to other causes (iron deficiency anemia, anemia of chronic disease, anemia due to B12 deficiency, anemia due to folate deficiency, anemia due to enzyme disorders, acquired and hereditary hemolytic anemias, aplastic anemia, and other nutritional anemias), patients who sustained complications of CPR (rib fractures associated with chest compressions and iatrogenic pneumothorax), and patients with a personal history of malignancy.

The primary outcome that was studied was in-hospital mortality, identified by the variable 'DIED' from the NIS database. Secondary outcomes studied included length of stay, pulmonary hypertension, pneumonia, renal failure, and seizures.

Data processing and statistical analysis was done using the SPSS version 26 software package (IBM Inc., Armonk, US). Age-adjusted mortality rates for each NIS year were calculated by utilizing US census data for each particular year as a standard population. Categorical data was compared using Pearson's chi-squared test, and continuous variables were compared using the independent samples t-test. Binomial logistic regression analysis was used to study the effects of sickle cell anemia on post-arrest in-hospital mortality with dichotomous variables, including medical comorbidities, and for the secondary outcomes analyses.

Multinomial regression analysis was used to study the effects of sickle cell anemia on post-arrest in-hospital mortality when variables had more than two independent values, including age subgroup analysis and demographic variables. Charlson comorbidity index scores were calculated to identify the burden of comorbid disease and were utilized as a potential confounder in multivariate regression analysis. The coefficients of correlation were calculated to study the secondary effects of sickle cell anemia on length of stay.

## Results

**Figure 1:**
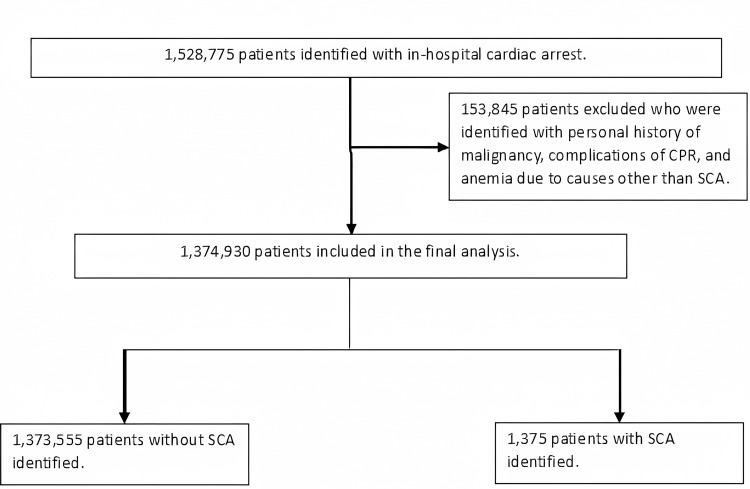
Flow chart demonstrating the study cohort CPR - cardiopulmonary resuscitation; SCA - sickle cell anemia

The primary inclusion criteria as identified by the methodology detailed above were met by 1,528,775 cases. Of these, 153,845 cases that met the exclusion criteria were excluded. The final analysis included 1,374,930 cases (Figure 1).

**Figure 2:**
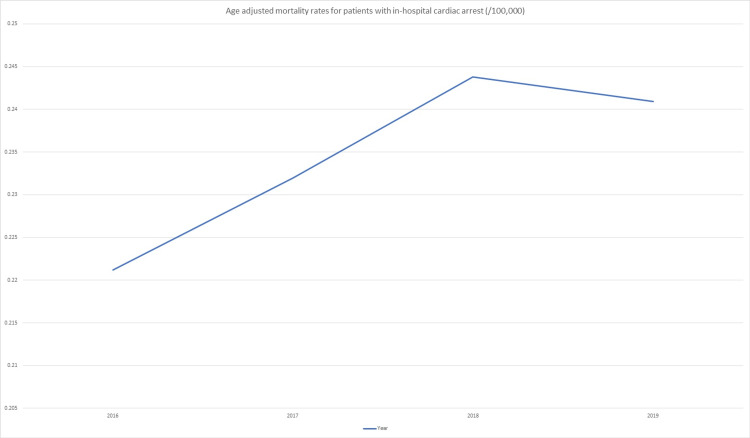
Age-adjusted mortality rates for patients with in-hospital cardiac arrest reported per 100,000 population

Age-adjusted mortality rates were calculated for the determination of all-cause mortality in the subset of patients who underwent an in-hospital cardiac arrest event for each year from 2016 to 2019. The results are represented graphically in Figure 2. A 10.2% increase in the mortality rate between the years 2016 to 2018 was noted, with a slight reduction in the mortality rate between the years 2018 and 2019 of 1.2%. The overall trend for the four years studied was one of increased mortality (Figure 2).

The baseline characteristics of this cohort have been listed in Table 1. Unadjusted for other factors, IHCA patients with SCA had a higher in-hospital mortality as compared to patients without SCA (32.7% vs. 21.5%). Interestingly, a higher percentage of the cohort with SCA were female as compared to patients without SCA (60.4% vs. 33.9%). A higher percentage of patients with SCA who had an IHCA also had Medicaid as their primary payer compared to patients without SCA (22.9% vs. 10.3%). A large racial discrepancy was noted among patients with SCA with an IHCA, who were noted to be a predominantly Black population (87.3%), as compared to patients without SCA with IHCA, who were noted to be predominantly White (73%). With regards to medical comorbidities, medical conditions that were more frequently identified in the study group as compared to the control group included congestive heart failure (48% vs. 37%), rheumatologic disease (6.9% vs. 3%), renal disease (40.7% vs. 24.6%), moderate to severe liver disease (12% vs. 7.2%), AIDS/HIV (1.8% vs. 0.3%), pulmonary hypertension (21.1% vs. 10.1%), and epilepsy (7.3% vs. 2.8%). The study group also had a younger population as compared to the control group (median: 53 vs. 66) and a higher Charlson comorbidity index (74 vs. 47.8).

**Table 1:** Baseline characteristics of patients with in-hospital cardiac arrest (IHCA) with and without sickle cell anemia (SCA) Values reflect the number of patients identified within the corresponding category. IQR: interquartile range

Variable	Without Hx of SCA (n=1,373,555)	With Hx of SCA (n=1375)
Death during hospital stay (%)	295,525 (21.5)	450 (32.7)
Sex (%)		
Male	907,325 (66.1)	545 (39.6)
Female	466,015 (33.9)	830 (60.4)
Primary payer (%)		
Medicare	759,535 (55.4)	595 (43.3)
Medicaid	141,160 (10.3)	315 (22.9)
Private insurance	385,490 (28.1)	365 (26.5)
Self-pay	43,395 (3.2)	65 (4.7)
No charge	3415 (0.2)	0 (0)
Other	38,835 (2.8)	35 (2.5)
Race (%)		
White	963,570 (73)	60 (4.5)
Black	156,145 (11.8)	1170 (87.3)
Hispanic	111,910 (8.5)	50 (3.7)
Asian/Pacific Islander	41,590 (3.2)	5 (0.4)
Native American	7095 (0.5)	0 (0)
Other	39,740 (3)	55 (4.1)
Other characteristics		
Myocardial infarction (%)	308,370 (22.5)	125 (9.1)
Congestive heart failure (%)	507,755 (37)	660 (48)
Peripheral vascular disease (%)	216,245 (15.7)	110 (8)
Cerebrovascular disease (%)	142,680 (10.4)	120 (8.7)
Dementia (%)	38,010 (2.8)	20 (1.5)
Chronic pulmonary disease (%)	252,965 (18.4)	205 (14.9)
Rheumatologic disease (%)	40,700 (3)	95 (6.9)
Peptic ulcer disease (%)	17,150 (1.2)	15 (1.1)
Hemiplegia/paraplegia (%)	27,250 (2)	35 (2.5)
Renal disease (%)	338,170 (24.6)	560 (40.7)
Moderate to severe liver disease (%)	99,435 (7.2)	165 (12)
AIDS/HIV (%)	4110 (0.3)	25 (1.8)
Diabetes (%)	534,910 (38.9)	520 (37.8)
Pneumonia (%)	143,985 (10.5)	195 (14.2)
Pulmonary hypertension (%)	138,820 (10.1)	290 (21.1)
Epilepsy (%)	38,555 (2.8)	100 (7.3)
Age, median (IQR)	66 (57-74)	53 (38-64)
Charlson comorbidity index, median (IQR)	47.8 (16-74)	74 (16-95)
Length of stay in days, median (IQR)	7 (5-12)	7 (4-14)

In the cohort of patients who had an IHCA, patients with SCA were found to have a significantly increased risk of in-hospital mortality adjusted for age, race, primary payer status, sex, and comorbidity burden as identified by their Charlson comorbidity score (OR: 1.16, 95% CI: 1.02-1.32, p=0.025). Other factors that were identified as being a significantly higher risk for in-hospital mortality after adjusting for confounders included male sex (OR: 1.49, 95% CI: 1.47-1.51, p<0.001), self-pay status (OR: 2.14, 95% CI: 2.06-2.21, p<0.001), Medicaid payer status (OR: 1.69, 95% CI: 1.63-1.76, p<0.001), Medicare payer status (OR: 1.13, 95% CI: 1.1-1.17, p<0.001), a higher Charlson comorbidity score (OR: 1.48, 95% CI: 1.48-1.49, p<0.001), and Black race (OR: 1.92, 95% CI: 1.87-1.97, p<0.001). The results are summarized in Table 2.

**Table 2:** Odd's ratios generated from multivariate logistic regression showing the effects of studied variables on in-hospital mortality in patients with in-hospital cardiac arrest LL - lower limit; UL - upper limit

Variable	Odd's ratio by logistic regression	LL 95% CI	UL 95% CI	p-value
Charlson comorbidity score	1.483	1.477	1.488	<0.001
Male sex	1.493	1.479	1.507	<0.001
Primary expected payer (Medicare)	1.132	1.010	1.165	<0.001
Primary expected payer (Medicaid)	1.692	1.639	1.746	<0.001
Primary expected payer (Private insurance)	0.656	0.637	0.676	<0.001
Primary expected payer (Self-pay)	2.139	2.063	2.218	<0.001
Race (White)	0.690	0.672	0.708	<0.001
Race (Black)	1.918	1.866	1.972	<0.001
Race (Hispanic)	1.063	1.033	1.095	<0.001
Race (Asian/Pacific Islander)	0.885	0.854	0.917	<0.001
Race (Native American)	0.902	0.847	0.962	0.0016
History of sickle cell disease or trait	1.159	1.019	1.319	0.0251

Of note, among different age groups, patients with SCA were found to have a statistically significant increased risk of in-hospital mortality in the 81-90 age category as compared to other age groups (OR: 2.5, 95% CI: 1.20-5.21, p=0.014)

A subgroup analysis was conducted to determine the relative contributions of sickle cell disease and sickle cell trait to the risk of in-hospital mortality. After adjusting for individual medical comorbidities of the Charlson comorbidity index, only patients with sickle cell disease were found to have a statistically significant increased risk of in-hospital mortality in the cohort of patients who sustained an in-hospital cardiac arrest (OR: 4.41, 95% CI: 3.5-5.55, p<0.001), whereas the cohort of patients with sickle cell trait did not meet statistical significance (p=0.71). All other medical comorbidities factored in the binomial regression analysis also significantly increased the risk of in-hospital mortality, as shown in Table 3.

**Table 3:** Subgroup analysis detailing odd's ratios generated from binomial logistic regression analysis showing the effects of sickle cell disease vs. sickle cell trait on in-hospital mortality in patients with in-hospital cardiac arrest, amongst other selected medical comorbidities from the Charlson comorbidity index

Variable	Odd's ratio by binomial logistic regression	Lower bound of 95% CI	Upper bound of 95% CI	p-value
Sickle cell trait	0.972	0.837	1.129	0.709
Sickle cell disease	4.408	3.504	5.546	<0.001
Congestive heart failure	1.113	1.103	1.123	<0.001
Myocardial infarction	1.039	1.028	1.049	<0.001
Cerebrovascular disease	1.085	1.069	1.1	<0.001
Dementia	5.235	5.123	5.349	<0.001
Chronic pulmonary disease	1.530	1.514	1.546	<0.001
Rheumatologic disease	1.289	1.259	1.32	<0.001
Peptic ulcer disease	1.597	1.544	1.653	<0.001
Mild liver disease	3.166	3.083	3.251	<0.001
Hemiplegia or paraplegia	1.929	1.876	1.984	<0.001
Renal disease	2.012	1.993	2.032	<0.001
Moderate or severe liver disease	4.627	4.563	4.692	<0.001
AIDS/HIV	2.635	2.468	2.813	<0.001

A secondary outcomes analysis was conducted to study the effects of SCA on selected secondary outcomes, including length of stay, pulmonary hypertension, pneumonia, renal failure, and seizures in patients with IHCA. Patients with SCA who died during the hospitalization were found to have a significantly higher risk of pulmonary hypertension (OR: 2.05, 95% CI: 1.61-2.61, p<0.001) and renal failure (OR: 1.75, 95% CI: 1.43-2.13, p<0.001) as compared to patients who did not die during the hospitalization. Length of stay was found to be negatively correlated in patients with SCA and IHCA who died during the hospitalization (correlation coefficient: -2.01, 95% CI: -3.03- -0.1, p<0.001) and positively correlated in patients with SCA and IHCA who did not die during the hospitalization (correlation coefficient: 0.99, 95% CI: 0.34-1.63, p=0.003). The results are given in Table 4.

**Table 4:** Secondary outcomes analysis of pulmonary hypertension, pneumonia, renal failure, seizures, and length of stay for patients with SCA who sustained an IHCA Results are expressed using odd's ratios from binomial logistic regression analysis and Pearson's correlation coefficients for the length of stay. HTN - hypertension; SCA - sickle cell anemia; IHCA - in-hospital cardiac arrest; LL - lower limit; UL - upper limit

	Pulmonary HTN	Pneumonia	Renal failure	Seizures		Length of stay
Died during hospitalization						
Odds ratio	2.05	0.69	1.75	0.78	Correlation coefficient	-2.01
95% LL	1.61	0.54	1.43	0.5	95% LL	-3.03
95% UL	2.61	0.88	2.13	1.23	95% UL	-0.1
p-value	>0.001	0.26	>0.001	0.28	p-value	>0.001
Did not die during hospitalization						
Odds ratio	1.79	1.72	2.11	4.22	Correlation coefficient	0.99
95% LL	1.51	1.4	1.83	3.35	95% LL	0.34
95% UL	2.11	2.1	2.43	5.32	95% UL	1.63
p-value	>0.001	>0.001	>0.001	>0.001	p-value	0.003

## Discussion

A single point mutation of the 7th amino acid of the beta-globin gene, valine for glutamic acid, causes the red blood cells to become rigid and sickle-shaped in low-oxygen environments. The combination of two α-globin and two mutated β-globin subunits creates hemoglobin S (HbS). The sickled red blood cells can lead to vascular occlusion and hemolysis. Homozygous alleles cause SCA, while heterozygous alleles cause sickle cell trait [2].

The present study demonstrates that patients with SCA do have an increased risk of in-hospital mortality after sustaining an in-hospital cardiac arrest (IHCA) after controlling for numerous confounders. SCD patients undergo recurrent episodes of ischemia-reperfusion injury to vital organs over time; thus, they are at an even higher risk of sustaining end-stage organ damage and multisystem organ failure post-IHCA. Chronic anemia from SCD leads to cardiac chamber dilation, an increase in left ventricular mass, and eventual left ventricular diastolic dysfunction [8]. Owing to how just under 50% of IHCAs are caused by preexisting cardiac conditions [8], cardiomyopathy, with and without pulmonary hypertension, may pose a risk to SCD patients during a crisis and especially in the post-IHCA setting. 

The commonest causes of death in patients with sickle cell disease were found to be cardiac (31.6%), respiratory (28.1%), renal (16.4%), infectious (14.4%), neurologic (11.9%), and gastrointestinal/hepatobiliary (9.2%) in etiology [9]. In previous decades, the most common cause of death was infection (33-48%) [10], and improved infection control practices, such as antibiotic prophylaxis, have been credited for the reduction in infection as a cause of death. Other prognostic factors have been shown to influence survival after an IHCA, with factors such as male sex, age greater than 60, active malignancy, and history of CKD all portending a decreased survival [11]. Myocardial infarction, congestive heart failure, renal disease, liver disease, dementia, and chronic pulmonary disease were all found to be significantly associated with increased in-hospital mortality in this study as well. 

The largest confounders in the study results were the Black race and self-pay status. SCA was an adaptive genetic evolution against severe malaria infection in African equatorial regions, with high-coverage sequencing and genotype data having confirmed the single African origin of the sickle-cell gene variant [12]. There are substantial racial differences in education, marginalization, and overall health and insurance coverage which create multidimensional poverty, and due to lack of education and/or funds, minorities such as African Americans and Latinos often utilize self-pay or Medicaid [13]. Patients at hospitals with lower cardiac arrest incidence rates were also found more likely to be white, have shockable rhythms, and be less likely to have renal, hepatic, and respiratory insufficiencies [14]. 

A disproportionate fraction of patients who sustain an IHCA with a history of SCA were found to be female as compared to male, despite which the male sex was still found to be significantly associated with in-hospital mortality. Studies have found a higher morbidity rate and a lower median age of death in adult males as compared to females [4], which has been attributed to hormone differences after puberty, as the mortality rate differences are not as significant before puberty. Estrogens may have a protective effect on endothelial functioning, including nitrous oxide bioavailability and functioning [15]. A prior study investigating mortality trends in sickle cell disease patients also found death rates to be higher among males despite a higher incidence of patients who sustained IHCA with a history of sickle disease being female [3].

Sickle cell trait (SCT) individuals only have one abnormal hemoglobin gene as compared to sickle cell disease, which has both hemoglobin genes that are abnormal. These individuals have a life expectancy similar to that of an individual without any hemoglobin deficits [16]. Studies have shown that patients with SCT have a general health comparable to that of patients without SCT [17], with a similar incidence of common medical conditions in both groups being reported. Additionally, although vaso-occlusive phenomena have been reported in SCT, the incidence of these phenomena is low compared to SCD [18, 19]. We found that although individuals with SCD have an increased risk of death after adjusting for comorbidities, patients with SCT did not. Similar findings have been reported in a large population study of US Army soldiers with SCT, and no increased risk of mortality was found [20]. 

Our study has considerable limitations that need to be accounted for. Coding errors and omissions may be present as we utilized an administrative database, and important patient information such as medication use, laboratory results, provider-dependent factors, and performance status of the patients are not available. Factors that directly affect patient outcome, such as the proximate cause of death, the effect of drug interactions, and non-procedural interventions performed, are not accounted for by this database. Confounding variables that are inadequately captured by this database aside from the comorbidity measures and factors accounted for in this analysis may have disproportionately affected the outcome, such as the proximate cause of the in-hospital cardiac arrest, patient frailty, prior treatments received, and withdrawal of care during hospitalization due to patient preferences.

## Conclusions

There is a significant difference in in-hospital mortality between those who have and those who do not have SCD in patients who develop IHCA. Our data also suggests different outcomes for patients based on ability to pay and race, with self-pay and Medicare/Medicaid patients and Black patients having an increased risk of in-hospital mortality compared to private insurance patients and White patients. While some of these allude to systemic issues as well as biological differences, it is important to consider why these trends occur and ways that these differences can be combated. Interestingly, our data also show that patients with sickle cell disease (HbSS) have a statistically significant increased risk of in-hospital mortality after IHCA (after adjusting for other medical comorbidities), whereas patients with sickle cell trait (HbAS) do not.
